# From a developmental atlas to maps of disease origin

**DOI:** 10.1002/ctm2.70790

**Published:** 2026-08-25

**Authors:** Jiexue Pan, Zhongliang Lin, Junlin Feng, Gaochen Zhang, Zejun Fu, Hefeng Huang, Guolian Ding

**Affiliations:** ^1^ Institute of Reproduction and Development Shanghai Key Laboratory of Reproduction and Development Obstetrics and Gynaecology Hospital Fudan University Shanghai China; ^2^ Shanghai Key Laboratory of Female Reproductive Endocrine Related Diseases Shanghai China; ^3^ Institute of Medical Genetics and Development Key Laboratory of Reproductive Genetics (Ministry of Education) and Women's Hospital Zhejiang University School of Medicine Hangzhou China; ^4^ Department of Obstetrics and Gynaecology Centre for Reproductive Medicine the Fourth Affiliated Hospital of School of Medicine, and International School of Medicine International Institutes of Medicine Zhejiang University Yiwu China

Many congenital disorders and diseases that present later in life may originate during embryonic development, long before structural abnormalities or clinical symptoms become detectable. Genetic testing can identify aneuploidies, copy‐number changes and pathogenic variants, but it often remains difficult to determine how a genetic lesion perturbs a developing human tissue and why the same genomic alteration can produce different organ involvement or disease severity. Interpreting pathology therefore requires a sufficiently detailed definition of normal development.

The recently established spatiotemporal transcriptomic atlas of post‐gastrulation human embryos provides such a reference. By profiling 77 sagittal sections from 13 euploid embryos spanning Carnegie stages 12–23 and integrating Stereo‐seq with single‐nucleus RNA sequencing, the study resolved transcriptional programmes across 50 organs and 198 substructures. Its translational value (Figure 1) may lie less in immediate clinical prediction than in providing a spatiotemporal coordinate system against which abnormal development can be measured, and in which the earliest deviations leading to disease can be located.^1^

**FIGURE 1:**
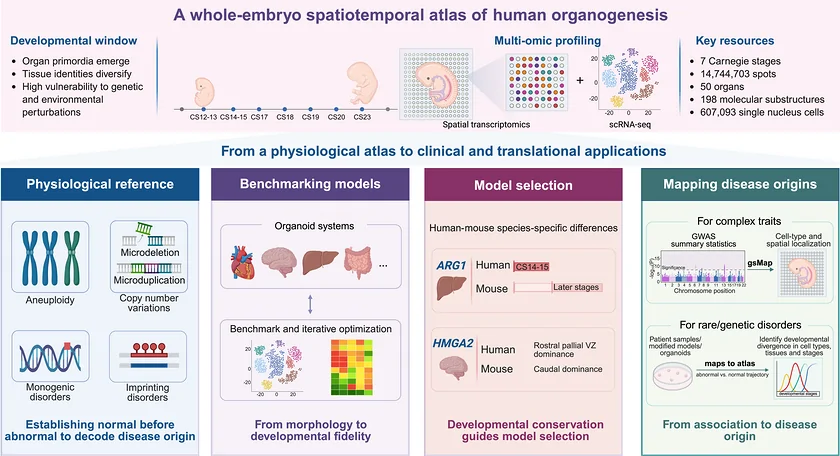
From a physiological atlas to clinical and translational applications.

## 1 A PHYSIOLOGICAL REFERENCE FOR DEVELOPMENTAL DISEASE

The biological consequence of a genetic abnormality depends on more than the variant itself. Gene dosage, developmental timing, cell identity, tissue interactions and parental origin can all shape the phenotype. The atlas mapped 1922 developmental disease‐associated genes and showed organ‐restricted, shared and broader multi‐organ expression patterns. It also identified spatially resolved allelic imbalance, adding another layer to the study of dosage and parent‐of‐origin effects.^1^

Recent disease studies illustrate why this normal reference matters. In trisomy 21, the pathogenic effect is not simply a uniform increase in chromosome 21 gene expression. Human iPSC and perturbation studies identified HMGN1 as a dosage‐sensitive regulator of atrioventricular cardiomyocyte states,^2^ while a recent single‐cell study of the developing Down syndrome cortex identified BACH1, PKNOX1 and GABPA as dosage‐sensitive regulatory hubs.^3^ In monosomy X, transcriptomic analysis of foetal tissues showed tissue‐dependent consequences involving both pseudoautosomal genes and genes escaping X inactivation.^4^ Evidently, the path from a fixed genomic abnormality to a variable clinical phenotype is not linear, but mediated by the changing landscape of gene regulation across different cell lineages and gestational windows. These findings support viewing chromosomal disorders as stage‐ and tissue‐dependent disturbances of developmental programmes.

Spatial context is equally important for microdeletion and single‐gene disorders. In 22q11.2 deletion syndrome, TBX1 is not strongly expressed in the cardiac neural crest cells that contribute to affected structures. Instead, Tbx1‐dependent signalling from adjacent pharyngeal mesoderm influences neural crest development.^5^ A whole‐embryo reference therefore preserves developmental fields and neighbouring tissues that may be missed when disease genes are interpreted only within the final affected organ. This underscores the necessity of a whole‐embryo perspective to uncover the indirect, non‐cell‐autonomous effects of developmental genes.

## 2 A BENCHMARK FOR DEVELOPMENTAL MODELS

Organoids and stem‐cell‐derived embryo models are increasingly used to study human development and disease, yet fidelity remains a central challenge. Different protocols can generate different cell compositions, maturation states and off‐target populations. An integrated human neural organoid atlas combining 36 datasets from 26 protocols demonstrated substantial protocol‐dependent variation and used primary developing brain datasets to quantify fidelity.^6^ A similar atlas of endoderm‐derived organoids integrated more than 800 000 cells across nine tissues and again highlighted the need for comparison with primary human tissues.^7^

There is no single universal metric that establishes whether an organoid faithfully represents a developing organ. Morphology, marker expression and selected functional readouts each capture only part of developmental fidelity. A human embryo atlas could add a common reference by asking whether a model matches the appropriate developmental stage, contains expected cell states and reproduces their spatial relationships and regulatory programmes.^8^ Prenatal organoids derived from amniotic fluid have already recapitulated selected features of congenital diaphragmatic hernia, illustrating how patient‐specific foetal models may be studied within clinically relevant developmental windows.^9^

## 3 CHOOSING MODELS BY DEVELOPMENTAL CONSERVATION

The atlas also provides a human developmental reference for evaluating the suitability of disease models. Cross‐species comparisons revealed differences in developmental timing and tissue distribution for several disease‐related genes, including *ARG1* and *CCBE1*.^1^ Spatial divergence was also evident in the developing brain, where *HMGA2* was enriched rostrally in the human pallial ventricular zone but showed a more caudal distribution in mice. These findings suggest that the presence of an orthologous gene alone does not ensure equivalent developmental context across species.

For strongly conserved developmental programmes, rodents remain powerful systems for causal and whole‐organism studies. When the relevant cell state, timing or regulatory programme diverges, human iPSC‐derived models and organoids may be more informative. Non‐human primate models may be considered for selected questions requiring intact physiology and primate‐specific development, but only when simpler systems cannot address the question. The atlas may therefore help establish an evidence‐based hierarchy of models rather than a one‐model‐fits‐all strategy.

## 4 MAPPING WHERE DISEASE BEGINS

A further opportunity is to connect developmental atlases with human genetics. In 2025, gsMap integrated GWAS summary statistics with spatial transcriptomics to localise cells and regions associated with complex traits.^10^ The method was tested with embryonic spatial datasets and applied to a Carnegie stage 8 human embryo.^10^ Applying similar approaches to the CS12–23 embryos could extend genetic association from a locus or cell type to a specific developmental region and stage.

This strategy is particularly relevant to complex diseases, for which genetic risk may act through transient progenitors long before the affected adult tissue is fully formed. Rare monogenic disorders and chromosomal abnormalities will require a complementary approach. Disease tissues, patient‐derived organoids or engineered models could be projected onto the normal atlas to identify the earliest point at which their developmental trajectories diverge.

Such maps may also refine how therapeutic targets are conceived. The primary genetic lesion may not itself be modifiable, but a downstream developmental programme could be. Experimental normalisation of dosage‐sensitive regulators in trisomy 21 has partially corrected molecular or developmental abnormalities in model systems.^2^, ^3^ While such findings remain confined to model systems, they demonstrate that identifying the critical developmental node—rather than the primary lesion—can uncover plausible avenues for early intervention.

## 5 REQUIREMENTS FOR TRANSLATION

Substantial work is required before developmental atlases can influence clinical care. On the technical front, the current reference is based on a limited number of euploid embryos and primarily captures transcriptional output in two‐dimensional sections. Larger and more diverse normal datasets, denser stage coverage and three‐dimensional reconstruction are needed. Spatial epigenomics, proteomics, metabolomics and lineage information will be required to distinguish markers of development from mechanisms that drive it. Moreover, future disease‐specific atlases should encompass chromosomal disorders, recurrent microdeletion and microduplication syndromes, imprinting disorders and well‐defined monogenic diseases, ideally extending to the placenta and maternal‐foetal interface.

On the validation and translational front, atlas‐derived hypotheses will require rigorous testing through perturbation and rescue experiments in model systems, followed by linkage to prenatal imaging, clinical phenotypes and long‐term postnatal outcomes. It is hoped that these efforts will ultimately bridge the gap between molecular trajectories observed in situ and the heterogeneous clinical presentations seen in patients.

The current atlas is not a diagnostic or prognostic tool. Its deeper clinical significance lies in establishing a normal developmental coordinate system. As disease maps, organoid benchmarks, human genetics and perturbational models become connected to this reference, the field may move from identifying genetic abnormalities to determining where and when development first deviates and which biological processes remain amenable to modification.

## CONFLICT OF INTEREST STATEMENT

The authors declare no conflict of interest.
